# Final‐year nursing students' placement experiences in a critical care setting: A qualitative study

**DOI:** 10.1111/nicc.13286

**Published:** 2025-02-19

**Authors:** Mary Edmonds, Naim Abdulmohdi

**Affiliations:** ^1^ Faculty of Health, Medicine and Social Care Anglia Ruskin University Cambridge UK; ^2^ School of Nursing and Midwifery, Faculty of Health, Medicine and Social Care Anglia Ruskin University Cambridge UK

**Keywords:** clinical experiences, clinical practice, critical care placements, nursing education, nursing students

## Abstract

**Background:**

Traditionally, ward placements have been utilized for nursing students to refine their management skills before registration. Whilst intensive care units (ICUs) offer unique learning opportunities, they are underutilized for student practice, and limited research has examined ICUs as conducive environments for developing management skills.

**Aim:**

This qualitative study aimed to explore the experiences of nursing students during their final‐year placement in critical care.

**Study Design:**

An inductive methodology was employed to explore the complexities of being a final‐year nursing student in critical care setting. Ten students were recruited between September 2019 and February 2020. Data were collected using individual, face‐to‐face semi‐structured interviews before the Covid‐19 pandemic. Thematic analysis was used to analyse the interview data.

**Results:**

Three themes were identified: barriers to learning, empowering transformation and a state of readiness. All the nursing students valued a placement that provided ‘real learning’ experiences and informed their needs and growth. Coaching and constructive feedback provided crucial markers and influenced students' learning performance. As their confidence increased, students became proactive in identifying complex caring activities to test and refine their knowledge and skills. Students visualized themselves working as a registered nurse within the critical care team.

**Conclusions:**

With appropriate support and supervision, a critical care placement for final‐year nursing students is a stimulating and transformational experience.

**Relevance to Clinical Practice:**

This study highlights the importance of critical care placements for nursing students, aiding their transition to professional practice. Integrating well‐supported placements into nursing curricula enhances decision‐making abilities and readiness for future roles. Critical care nurse educators and managers should focus on developing effective support systems during these placements. By providing tailored support and preparing students for the demands of critical care, these experiences can improve job satisfaction and confidence, which are crucial for staff recruitment and retention in this challenging field.

**What is known about the topic**

- Ward placements traditionally developed nursing students' management skills before becoming registered nurses.
- Intensive care units (ICUs) continue to be underutilized for student practice in developing management skills, with limited research in this context.

**What this paper adds**

- Highlights that critical care placements aid final‐year nursing students' transition to professional practice
- Nursing students expressed that well‐supported critical care placements can enhance their skills, decision‐making and readiness for their future roles.

## 1 INTRODUCTION

The shortage of qualified critical care nurses poses a risk to patient safety and delivering high‐quality patient care. Despite an increase of nursing graduates in recent years, the number of newly qualified nurses continuing to leave the profession remains high, further exacerbating challenges with staffing turnover.^1^ A meta‐analysis by Xu et al.^2^ revealed that 27% of critical care nurses globally planned to leave the profession. In the UK, many critical care units experience annual staff turnover rates exceeding 20%, with some reaching as high as 42%.^3^ Additionally, a recent survey by the UK Critical Care Network (CC3N) found that half of the current adult critical care nurses expect to leave their positions within the next 3 years.^4^ Research has shown that low critical care nurse staffing levels are linked to omissions in essential nursing care and adverse patient outcomes, including increased mortality.^5^ Consequently, maintaining adequate staff‐patient ratios is crucial for ensuring effective critical care services. Ensuring a positive experience in the final clinical placement could be a key strategy in enhancing new nurse graduates' transition to their registered nurse role, increasing retention and strengthening commitment to the nursing profession.^6^

Clinical experiences are a crucial component of nursing education as they allow the nursing students to apply theoretical knowledge in real‐world settings. Providing nursing students with quality placements is an important necessity for both education institutions and practice providers, involved in the preparation of the future nurse. Clinical practice fosters the development of technical skills, clinical reasoning, professional attributes and the achievement of nursing proficiencies across various clinical contexts.^7^ Additionally, these clinical experiences provide an ideal opportunity for students to engage in self‐reflection and enhance their communication and teamwork skills through interactions with patients, their families and multidisciplinary healthcare teams. The final placement before graduation is a pivotal phase in nursing education, shaping students' professional identities and commitment. It bridges theory and practice, exposing students to real healthcare challenges and values. This period aids their transition to registered nurse, helping them discover their nursing specialty and instilling dedication.^6^

Intensive care units (ICUs) provide unique learning opportunities in technical skills, interdisciplinary learning and patient communication. Despite challenges such as stress related to complex cases,^8^, ^9^ students generally enjoyed their ICU clinical experience, appreciating the quality of the learning environment compared with other settings.^7^ These units are highly complex, with the added pressure of caring for critically ill patients, which can impact students' learning experiences.^10^ Consequently, ICUs are not commonly used for student practice during their final‐year clinical management placement. The complexity of the healthcare system demands that nurse graduates are well‐prepared to handle demanding situations, assume clinical leadership roles and manage complex clinical encounters. Critical care placements offer nursing students opportunities to adapt to changing needs in 21st‐century healthcare. Different countries with varying educational systems reflect differences in when nursing students experience a critical care placement. For example, in the UK, Conneely and Hunter^11^ reported placements in the first year, whilst in Canada^12^ and Turkey,^13^ placements in critical care occurred in the second year. Other studies reported that students attended critical care placements in their fourth year in both Australia^14^ and Turkey.^15^, ^16^

However, doubt about the suitability as a final placement before graduation has meant critical care areas are being underutilized. Given that the purpose of a final placement is to develop and enhance leadership, patient management, prioritization, time management and communication skills, the rationale is unclear. This provided the catalyst for one critical care unit in a large cardio‐thoracic hospital to enable final‐year nursing students to have a placement, at a time when no other critical care units in the local vicinity received final‐year nursing students. This research was conducted to explore the experiences of students who attended final management placements in critical care units, contributing to the literature in an area that has been understudied.

## 2 RESEARCH AIM

The purpose of this qualitative study was to explore the placement experiences of final‐year nursing students in one critical care setting, understand how these students coped during their final‐year placement and the contribution this clinical placement had upon their learning as a final‐year nursing student.

## 3 DESIGN AND METHODS

This study adopted an inductive methodology and a descriptive qualitative design^17^ seeking to understand the complexities and realities of being a final‐year nursing student in a critical care setting. Ten students were recruited between September 2019 and February 2020. Data were collected through individual, face‐to‐face semi‐structured interviews, conducted in a pre‐booked room prior to the Covid‐19 pandemic. The interview data were inductively analysed using thematic analysis based on Braun and Clarke.^18^

### 3.1 Setting

The cardio‐thoracic critical care unit in England, in which this study was conducted, had a bed capacity of more than 40 beds. It provides expert care for adult patients with a range of cardiac, thoracic and vascular problems, major cardio‐thoracic and transplant surgeries. Nursing students participated in a 24‐h cycle of care and worked with expert multidisciplinary teams. The clinical placement lasted 12 weeks and provided exposure to a wide range of caring situations and managing families in vulnerable states. Critical thinking and analysis of patients' physiological status is required by students. A qualified critical care nurse was designated to work every day with each nursing student to guide them through their experiences.

### 3.2 Participants

A convenience sample of final‐year nursing students enrolled in the Bachelor of Science (BSc) program at a university in England, and who had completed a clinical placement in a critical care setting, were recruited. Each nursing student had either completed or were close to the completion of a 12‐week core placement (the last 3 months of their programme). The timing of recruitment was important to ensure they had first‐hand experience of a range of activities within their final placement in a critical care setting. An invitation was sent to all students in those cohorts through their email and during an in‐person session with the course leader. Those interested in participation in this study contacted the first author (ME). A total of 10 nursing students (five female and five male) agreed to participate between September 2019 to February 2020. This sample originated from three different cohorts within the same university, each with a different start time. Recruitment of students occurred at the same time point of their programme from three consecutive cohorts. At the time of this research, authors (ME and NA) were not directly involved in teaching or assessing the participants in this study but had responsibility on supporting them in clinical placement as link educators to the hospital.

### 3.3 Data collection

Individual semi‐structured interviews took place in a pre‐booked room at the university campus when the nursing students were off duty. Semi‐structured interviews were conducted by the first author using an interview guide. The interview guide was pilot tested during the first interview, and one minor correction was made.

Each interview took between 12 and 20 min, was audio recorded and may have contained the following questions:

- Can you tell me what is it like being a final‐year nursing student in the critical care setting?
- What types of caring activities have you participated in as a final‐year nursing student?
- How do you feel these have helped your management skills?
- Is there anything that has made it difficult to undertake management skills in critical care?
- Have you found it a stressful environment as a final‐year placement?
- Who supported your learning in the setting?
- What made a difference to your experiences?
- Have you found critical care to be a good placement for final‐year nursing students?
- Is there anything else, you think would be useful to share with me?

### 3.4 Data analysis

An inductive approach was used to analyse the content of the interviews. Thematic analysis is a method for identifying, analysing, organizing, describing and reporting themes found within a data set.^18^ The interviews were transcribed by the first author and then thematically analysed following the steps described by Braun and Clarke.^18^ The transcripts were read in one session to develop familiarity of the data. This step was repeated several times to immerse and enhance the trustworthiness of the analysis. To further support the credibility of the research and conclusions, authors (ME and NA) independently conducted data analysis. The first author used a paper‐based approach, and the second author used the NVivo 12 software for data analysis. The findings from both approaches were discussed and agreed. All transcripts were coded in a systematic way generating a list of descriptive codes. The codes were then clustered together to identify patterns. All the sub‐themes were aggregated to illustrate a smaller number of meaningful patterns that were considered the main themes. Analysis focused on emergent themes which addressed the primary research questions. The authors stopped data collection when no further new information was identified.

### 3.5 Ethical considerations

Participants were informed their identity would be pseudonymised in any conference proceedings or publications arising from the study. Participation was voluntary. All participants were given a participant information sheet. Students were invited to participate via the programme lead who informed the students about the purpose of the research. This was to ensure nursing students did not feel pressurized to participate. The University Research Ethics Committee approved this study (approval number FHSCE‐DREP‐17‐259 on the 17th of Sept 2018), and course leaders/senior managers at the University gave institutional approval. Participants' consent was obtained before the interview.

## 4 FINDINGS

Six participants (60%) were aged 21–30 years. The participants included five women, three of whom were from a British white background and two from a Black Caribbean background. Among the five male participants, one was from a British white background, whilst the remaining four were from any other ethnic minority group. Ethnicity grouping was based on the UK Office for National Statistics (2021) classification. Six participants (three females and three males) had previous clinical placements in critical care, and four of them were seeking employment in this area. In total, five participants were seeking employment in critical care, and of those, four were male (Table 1).

**TABLE 1:** Sample characteristics.

Characteristics		Number (%)
Age range (years)	20–25	4 (40)
	26–30	2 (20)
	31–35	1 (10)
	36–40	3 (30)
Sex	Female	5 (50)
	Male	5 (50)
Ethnicity	British white background	4 (40)
	Any other ethnic minority group	5 (50)
	Black Caribbean background	1 (10)
Previous clinical placement in critical care	Yes, in second year of their BSc degree	4 (40)
	Yes, in third year of their BSc degree	2 (20)
	No, never had placement in critical care	4 (40)
Intention to work in critical care immediately post‐qualification	Yes	5 (50)
	No	5 (50)

**FIGURE 1:**
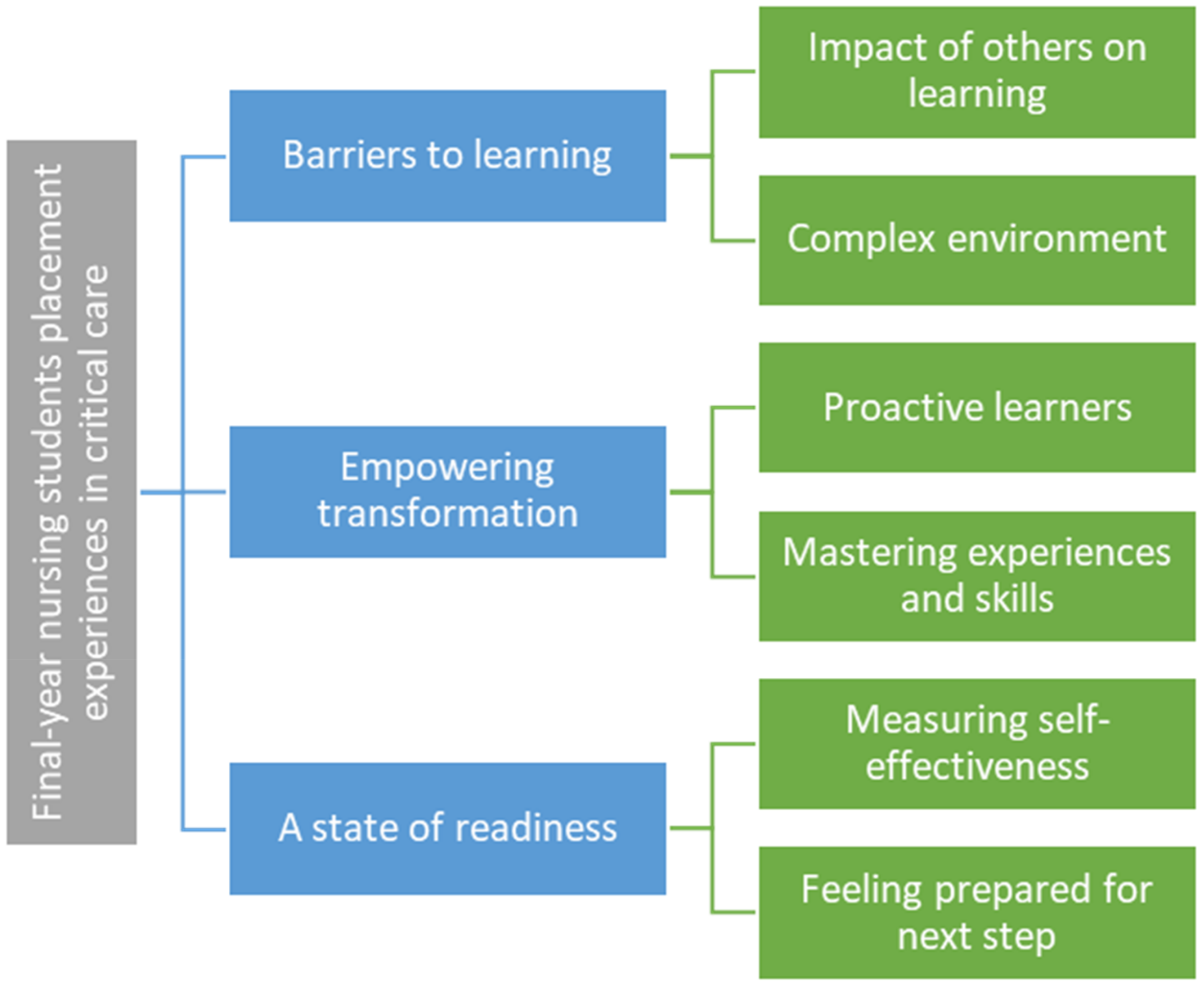
Themes.

The data was categorized into three themes: barriers to learning, empowering transformation and a state of readiness (Figure 1). Each theme had two sub‐themes. The students' real names are replaced with a pseudonym.

### 4.1 Theme one: Barriers to learning

#### 4.1.1 Impact of others on learning

Three nursing students commented on how they were stopped from participating in certain experiences because they were a student. This made them feel annoyed that learning opportunities had been limited.

I remember when I said I wanted to learn about ECMO [extracorporeal membrane oxygenation] and somebody said, you don't really have that much time to learn about this and you wouldn't get a chance to look after an ECMO patient, considering you are still a student, you would just be observing. Isaac.

Some staff were nice to have you and then they just spent the day zipping off doing everything and you would just be sat there going umm what's going on. Solomon.

In contrast, nursing students had to prepare themselves for a higher than anticipated level of knowledge. Whilst they recognized the benefits, they found the high levels of expectations made them feel nervous and inadequate.

In the first and second week it really affected me because as a final‐year nursing student they were expecting me to know a lot. It was frustrating at first because they were pushing me and giving me feedback that I needed to do more. I thought I had done well in my last placement, so why am I having this feedback? Someone was asking me about sodium and potassium, she expected me to know all of this but then I told her I was on my second week of my final placement. The level of knowledge they have, is very advanced. Kaseem.

#### 4.1.2 Complex environment

At the start of their placement, nursing students experienced apprehension and fear about caring for patients surrounded by unfamiliar machines and advanced monitoring systems, which reinforced the severity of the patients' conditions.

In the beginning it was quite scary because there's a lot of machines that I hadn't seen before and there were a lot of intravenous drugs which I hadn't had much practice with before. So, at the beginning, it was really quite scary and nerve racking because there is so many things going on at the same time. Anna.

It's scary, when you walk into a room and the bed is covered in machines and you think I don't even know how I am going to roll this patient. Blessing.

### 4.2 Theme two: Empowering transformation

#### 4.2.1 Proactive learners

Overall, the final‐year nursing students felt empowered to identify their own learning needs and find the information to manage new caring situations.

I went and read, just to have a bit of knowledge before the extubation and we managed to extubate the patient. It was very good. I never felt I communicated that well and I found here, I had to improve my communication skills. Isaac.

I could go in and think I haven't cared for a ventilated patient yet, is it okay if I go with a ventilated patient? Blessing.

Because it was only one patient, I had to think about the steps ahead and learn more clinical skills. Aaron.

Several of the nursing students commented positively on the support of the teaching team. This support helped the nursing students make sense of what they were doing and why.

The teaching team were really very good. We got to go to the new starters study days which helped with the theory behind the practical. It meant we could work with anyone, which meant we could pick our patients rather than working with the same nurse and having the same patient. Blessing.

I am going to management meetings and business meetings. These helped me to understand the pressures of critical care and why patients need to be stepped down. Natalia.

Everything is planned. One day you are on shift, the next day you go with the multi‐disciplinary team. Rather than teaching, it is more like a discussion, they all try to get the best from you. Alex.

#### 4.2.2 Mastering experiences and skills

Managing deteriorating sick patients presented the final‐year nursing students with multiple opportunities and skill sets to acquire. These situations included head to toe assessment, communication with the wider team, application of existing knowledge, medications and being hands on.

You are not managing a whole bay of patients like you are on a ward, but you are managing a patient who is potentially deteriorating or even if they are not deteriorating and their getting better; they still have very high requirements. You are managing the decisions. Are we going to wean this down? Should we get the physiotherapists involved and try to get them out of bed? There is no less management on a critical care than there is on a ward, it is just a very different type. Constance.

In critical care you are looking after and managing one patient and communicating with the family. I was handing over and talking about the whole case from the beginning with the history of the patient and everything. Aaron.

One nursing student expressed how being competent did not necessarily reflect confidence but the input from those supporting her learning was important for regulating her inner self.

The team assess how responsible you are and how quick at reacting you are because it is the place where you must save lives and lots of emergencies happening during the day. You know you are going to be alone one day; you are going to be responsible, and you can believe in yourself. I am not saying I had 100% confidence, but I did a lot of inside learning. Jade.

### 4.3 Theme three: A state of readiness

#### 4.3.1 Measuring self‐effectiveness

The enhancement of skills and knowledge exerted a positive influence on nursing students' recognition of their personal development and growth during the placement.

I feel good because I have awareness of what the MDT does because if your patient has got a problem, it's your responsibility, you need to know who to call and how it works. Constance.

If you can deal with the exposure you get on critical care, you can deal with anything. Whether it is just keeping a patient stable or getting them better, if you can deal with those situations then you will be able to deal with absolutely anything and having had my final placement there, I really do feel that confidence and that ability just to deal with anything now. Solomon.

Now I take more control. I manage my patient, speak to doctors, try to do everything myself rather than relying on the nurse to tell me or do it for me. Natalia.

#### 4.3.2 Feeling prepared for next step

There was an acknowledgement and understanding of what being a registered nurse could provide. This placement had provided the foundations for the next steps.

I have learnt a lot and I feel so much more confident. Every time I go in, I feel like it's a second home. I am now, let's get started and tell me what happened. Anna.

I can manage the patient using ABCDE [airway, breathing, circulation, disability and exposure], be hands on, know the equipment, ask what is happening with the patient. It's more than managing the patient, it makes you a whole nurse. Kaseem.

## 5 DISCUSSION

The findings of this qualitative study reflected how these 10 final‐year nursing students all valued the importance of having a placement in the critical care setting. The three themes generated from the nursing students' responses highlight the skills and practice learning that informed their learning needs and growth. A prominent message across all three themes was the strong belief that their knowledge, skills and performance had been enhanced because of ‘real learning’ opportunities in this clinical setting. This made the nursing students feel ready and prepared for becoming a qualified nurse.

The critical care placement provided the nursing students with a wealth of opportunities to progress their learning. The final‐year nursing students described their participation in the care of cardio‐thoracic‐surgical patients, weaning patients from ventilators, management of life‐threatening arrhythmias, extubation, management of complex machinery, arterial blood gas machines, ventilators and skills in monitoring the deteriorating patient, intravenous drugs, naso‐gastric tubes, assessment and haemodynamic monitoring. These different experiences stimulated their self‐evaluation of their abilities and effectiveness of their contributions. This is also consistent with a Turkish study conducted by Saglam et al.^9^; they also found students developing caring skills, a sense of responsibility, knowledge and competences during critical care placements.

The nursing students were proactive in identifying the types of clinical encounters and skills they needed to acquire so they could respond, act and manage a range of situations. They were keen to seek out the ‘right’ challenges and workload, reflecting a mature approach to their learning. These included tackling challenging tasks, trying things out, working out what had worked well and what needed further practice. Trying out things was a purposeful behaviour but also coupled with a level of risk, in that more learning was required or expected. By the end of the placement, the nursing students believed they had mastered, refined and extended their knowledge and skills for their level. Feeling more confident was also noted in research conducted in Canada,^12^ Australia^14^ and Turkey.^16^ On the spot questions from qualified nurses enabled existing and new knowledge to be tested and checked. Coaching and supervision helped the nursing students to reflect on current and onward learning trajectory. According to Eraut,^19^ these learning activities can occur many times and success depends on the available learning opportunities and the quality of relationships from those who are supporting nursing students.

All the nursing students agreed this critical care placement was conducive to an active and vibrant learning climate. Nursing students witnessed and worked alongside experienced clinical staff and participated in different kinds of knowledge and expertise. The skills they obtained were viewed as essential for working in a critical care setting with a recognition that these would differ to what was needed in a ward placement. An invitation to participate in the in‐house training usually given to newly qualified nurses provided explanations for clinical knowledge but also created a sense of social inclusion. The students felt part of the critical care team and that their contributions had been valued and accepted by the team. Similar findings were identified in Liu et al.'s^20^ qualitative meta‐synthesis.

The nursing students' understanding of their learning and performance was strongly influenced by their emotional constructs. Consistent with other studies, the final‐year nursing students felt overwhelmed and anxious at the start of their placement because of the complex nature of the environment.^8^, ^9^, ^12^, ^16^ However, this was short lived and related to being a newcomer. The length of the placement (12 weeks) also seemed to diminish these challenges.^9^, ^12^ Gradually as the nursing students felt more confident, they sought different situations to test and enhance their knowledge and skills. Success in performing complex caring activities reinforced the nursing students' beliefs they had succeeded (problem‐solved). This enhanced their self‐confidence and motivation. Self‐efficacy beliefs, according to Bandura,^21^ are important for influencing behavioural changes and clinical performance. The mediating effects of these beliefs enabled the nursing students to see themselves as part of the critical care team. Their performance was further rewarded when clinical staff provided the students with different learning opportunities. It was important for these final‐year nursing students to be seen as able to care and manage challenging or complex situations because this reflected their growth and learning.

Feedback from others was a crucial process for the final‐year nursing students in terms of establishing a marker for where they had reached and determining which skills still needed to be acquired. Halcomb et al.^14^ and Zi‐Xiang et al.^22^ researched final‐year student nurses' experiences of receiving feedback and the importance for this to have purpose, structure and be timely. Whilst feedback could empower nursing students, it also had the ability to alienate, cause stress and disengagement. Critical feedback and anxiety about one's performance can influence the emotional dimension of a person's learning and working with others.^23^, ^24^ An important dimension that requires the supervisor and managers attention to provide adequate and timely support. According to Eraut,^25^ feedback given is not the same as feedback received. When the stakes are high, as perceived by final‐year nursing students, feedback can be viewed negatively, particularly when there is a suggestion for improvement. On occasions, this led to a self‐protecting behaviour, a critical questioning and/or doubt of others who may have confirmed their abilities in previous placements.

Whilst most learning occurred through the support of the critical care staff, there were occasions when the nursing students felt excluded. As newcomers, the nursing students had to make it explicit to the staff they needed their support to make learning opportunities available and/or participate in complex aspects such as caring for ECMO patients. Positive appraisal from nurses viewed as experts had a noticeable impact on the final‐year nursing students' self‐motivation and commitment. When students felt a sense of belonging within the qualified staffing team, they felt more competent in being able to manage complex patients. At the end of the placement in our study, students described a sense of anticipation and readiness for the next stage of their journey. This is consistent with the findings reported in Salem's.^15^ Students in our study imaged their future success as a registered nurse, able to prioritize workloads, manage complex situations and communicate to others; they had accomplished what was required for this milestone. Overall, the students' experiences demonstrate the benefits of having a critical care placement as a final management placement and its valuable role in shaping students' readiness for clinical practice.

The insights gained from this study on the benefits of critical care placements, along with the identified challenges, should inform the development of educational and supportive strategies. By addressing these findings, we can create more effective placement programmes that not only prepare future nurses for the demands of critical care but also support their well‐being and reduce the risk of burnout early in their careers. These strategies could contribute to building a resilient and sustainable critical care nursing workforce.

## 6 LIMITATIONS

This study gathered subjective data from final‐year nursing students who had either completed or were close to completing their final placement in one critical care unit. This was a self‐selecting sample of nursing students, and therefore, the results of the study are not generalizable. The 10 final‐year nursing students originated from three different cohorts because of the small numbers of students allocated to the one critical care setting in one hospital. The potential impact of being known to the author's and on the students' responses or the obligation to participate was acknowledged. In recognition that students' perspectives may have changed over time because of their different experiences, they were not asked to provide feedback on their transcripts and findings. However, the students' insights are transferable and can be used to inform and enhance the placement in a critical care setting for final‐year nursing students.

## 7 IMPLICATIONS FOR PRACTICE AND RESEARCH

This study emphasizes the significance of critical care placements for final‐year nursing students, facilitating their transition to professional practice. It underscores the value of coaching, feedback and proactive learning in preparing students for real‐world nursing practice. Integrating well‐supported critical care placements into nursing curricula can enhance essential skills, decision‐making abilities and readiness for future nursing roles. Critical care nurse educators and managers should focus on developing effective support systems during these placements. By providing tailored support and preparing students for the demands of critical care, these experiences can improve job satisfaction and confidence, which are crucial for staff recruitment and retention in this challenging field. More research is warranted to explore the experiences of nursing students allocated to different critical care placements. The sample could be gathered quantitatively over multiple time points to establish whether there are changes in their experiences. Data could be compared between critical care placements to discover gaps and similarities to provide a fuller understanding. Future research from the practice providers' perspective is also needed.

## 8 CONCLUSION

This study confirmed that a critical care setting has the potential to motivate nursing students to reach their desired goals and achieve the learning outcomes required for a final‐year placement. The findings revealed that nursing students acknowledged the value of the learning opportunities and support and commitment from the education team and nursing staff. The study's findings support the need for an overhaul in the perceptions of nontraditional final‐year placements to include settings such as critical care areas. At a time when placement provision for nursing students has seen significant changes because of Covid‐19, a positive learning experience can influence career choice, and the development of clinical skills and knowledge, preparing them to be Future Nurses.

## PERMISSION TO REPRODUCE MATERIAL FROM OTHER SOURCES

Permission to reproduce material from other sources was not required for this study, as no material from external sources was reproduced in the course of the research.

## Data Availability

The data that support the findings of this study are available from the corresponding author upon reasonable request.
